# Enhanced soil quality with reduced tillage and solid manures in organic farming – a synthesis of 15 years

**DOI:** 10.1038/s41598-020-61320-8

**Published:** 2020-03-10

**Authors:** Maike Krauss, Alfred Berner, Frédéric Perrochet, Robert Frei, Urs Niggli, Paul Mäder

**Affiliations:** Department of Soil Sciences, Research Institute of Organic Agriculture (FiBL), 5070 Frick, Switzerland

**Keywords:** Microbiology, Biogeochemistry

## Abstract

Demands upon the sustainability of farming are increasing in step with climate change and diversity loss. Organic farming offers a viable approach. To further improve organic management, three strategies with potential to enhance soil quality are being tested in a long-term trial since 2002 on a clay loam in temperate Switzerland: reduced tillage vs. ploughing, solid vs. liquid manures and biodynamic preparations. A synthesis of 15 years reveals an increase in topsoil organic carbon (SOC, +25%), microbial biomass (+32%) and activity (+34%) and a shift in microbial communities with conversion from ploughing to reduced tillage. Soils under reduced tillage are more stratified in SOC and nutrients. Additional application of composted manure has increased SOC by 6% compared to pure slurry application, with little impact on soil microbes. Biodynamic preparations have had a minor impact on soil quality. Fertilisation and biodynamic preparations did not affect yields. Both higher and lower yields were harvested in the reduced tillage system in relation to ploughing. The main yield determinants were N supply and higher weed infestation under reduced tillage. Continuously reduced tillage in organic farming has been proven to enhance soil quality at this site, while also presenting more challenges in management.

## Introduction

Various global challenges are calling for change. Soil erosion and degradation, loss of biodiversity and pollution of adjacent natural systems all call current agricultural management practices into question. To meet sustainability requirements, there is a special need to develop, beyond individual innovations, alternative systems that consider the surrounding environment.

Organic farming can serve as an alternative to conventional farming, as it has been shown to lower environmental impacts while being able to feed the world if consumption behaviour is adjusted^1^. Organic farming is based on a low external input strategy with legumes, green manures, wide crop rotations and organic fertilisation. All of those approaches build and maintain soil quality^2^. Biological mobilisation of soil nutrients for crop nutrition is more important in organic than conventional farming, meaning that soil quality needs to be maintained at a high level. Yet there are management practices and trends in organic farming that may jeopardise soil quality and hence require rethinking and improvement.

Regarding primary tillage, mouldboard ploughing is common in organic farming in Europe, where it is an important tool for weed control and organic matter management^3^. A bare soil surface is left after ploughing. This increases the risk of soil erosion and hence loss of fertile soil^4^. Ploughing is assigned to “conventional tillage” (CT) in the context of the present study. As an alternative, conservation tillage systems have been developed globally for nearly a century^5^. Conservation tillage includes “no-till” (NT) with direct drilling of seeds and various kinds of “reduced tillage” (RT) practices that are either defined as less intensive than conventional tillage^6,7^ or by the amount of residues left in the field (defined as 15–30% residue cover by CTIC^8^). No-till with herbicide application is widespread^9^ but increasingly under pressure due to the rise of herbicide-resistant weeds and health issues with regard to herbicide toxicity^10,11^. Nutrient stratification is a further problematic issue^11^. In Europe, many soils are contaminated with pesticides, especially with glyphosate and its metabolites^12^. In the light of calls for agricultural systems with lower environmental impacts, the following question arises: Is it possible to integrate conservation tillage into organic farming without herbicide application, and if so, then how? Some decades ago, research on this question started, often with a focus on reduced tillage systems where tillage operations work in shallower depths and mostly avoid soil inversion^6^. Today there is increasing knowledge of and interest in herbicide-free conservation tillage systems, including RT and NT^7,13^. Dedicated research in long-term trials as presented in this paper has contributed to this awareness and to a willingness to replace ploughing.

Another trend can affect soil quality: Over the last decades, the number of farms has decreased and herd sizes have increased^14^, which implies larger stables. Faeces are increasingly stored and applied as liquid slurries for a more efficient workflow. Opting for solid farmyard manure and especially composted manure is more labour intensive. Liquid fertilisers contain more available nitrogen that often converts into higher yields in the short term. However, solid manures are valuable fertilisers that serve to maintain or even increase soil organic matter and thus soil quality in the long run, as seen e.g. in the DOK trial which started in 1978 in Switzerland^15^ and in the long-term trial in Bad Lauchstädt in Germany which commenced in 1980^16^. Liquid versus solid fertilisation thus also reflects a different fertilisation strategy. The question here is whether fertilisation with liquid manures alone can maintain soil quality.

In both above-mentioned system comparison trials, systems that include composted solid manures and biodynamic preparations performed best in maintaining soil organic matter levels. Biodynamic preparations were hence found to be a technique used by farmers to improve soil quality. However, their impact is difficult to elucidate separately in a system comparison approach.

In Switzerland, a demand consequently arose among farmers and researchers in early 2000 to test an improved organic management regime with a focus on soil quality in a factorial field trial. The long-term trial in Frick started in autumn 2002. The reference system is Swiss organic management with ploughing and slurry fertilisation. Three improved management strategies were included in the trial: i) conservation or, in this case, reduced tillage (RT) instead of ploughing (CT), ii) mixed fertilisation with solid manure compost and slurry (MC) instead of slurry only (SL) and iii) additional application of biodynamic preparations (with vs. without). All strategies were hypothesised to increase soil quality while maintaining yields. The adoption of a continuous reduced tillage system was expected to be agronomically more challenging than ploughing. As soil quality is best monitored with a range of indicators^17^ we assessed soil chemical and biological indicators regularly.

This paper synthesises 15 years of research from autumn 2002 to 2018, including soil and agronomic data over three crop rotations and additional highlights from 14 peer reviewed publications on in-depth investigations. With our long-term research, we underscore the potential of improved organic management for soil conservation.

## Materials and Methods

### Site description

In Frick (Switzerland), a long-term trial was established at the farm of the Research Institute of Organic Agriculture (FiBL) in autumn 2002. At that time the farm had been managed organically for seven years according to European Union Regulation (EC) No. 2092/91 (later No. 834/2007) and was certified to the Bio Suisse Standards, with dairy cattle and pig breeding and an arable-ley rotation ploughed to 15–20 cm. Previously, the farm had been conventional with livestock and an arable-ley rotation ploughed to 25 cm. In 2010, the farm was converted to biodynamic farming (Demeter certification) with dairy milk and cheese production. The trial site (47 °51′20′′N, 8 °02′36′′E, 350 m altitude) is situated on a clay loamy soil, a stagnic eutric Cambisol. It consists of 45% clay, 33% silt and 22% sand. It had 2.2% soil organic carbon (SOC) and a pH of 7.1 (H_2_O) in 2002. The soil was rich in available P and K at trial start. Details can be found in Berner, *et al*.^18^. The annual average temperature and precipitation in 2003–2018 was 10.4 °C and 1030 mm respectively. The climate diagram is shown in Fig. S1 Supplement.

### Experimental setup and management

The three factors – tillage, fertilisation and biodynamic preparations – were arranged in a strip-split plot design. These eight treatments were replicated four times to total 32 plots of 12 × 12 m size that allow for management with normal farm machinery. Block distribution accounts for the spatial gradient in clay content. The crop rotation was modified slightly over the years with silage maize (*Zea mays* L., cv. Amadeo), winter wheat (*Triticum aestivum* L., cv. Titlis), an oat-clover-vetch intercrop (*Avena sativa* L., *Trifolium incarnatum* L., *Vicia villosa* R.), sunflower (*Helianthus annuus* L., cv. Sanluca in 2004, Delfi in 2010), spelt (*Triticum spelta* L., cv. Ostro) and a 2-year grass-clover ley mixture (*Trifolium pratense* L., *Trifolium repens* L., *Lolium perenne* L., *Festuca pratensis* Huds., *Dactylus glomerata* L., *Phleum pratense* L.) in the first two rotations. In the third rotation, winter wheat was grown after grass-clover to facilitate ley termination in the reduced tillage plots in late summer and to adjust for the new rotation of the FiBL farm. The third rotation accordingly consisted of winter wheat (cv. Wiwa), silage maize (cv. Fabregas), spelt (cv. Ostro) and two years of grass-clover (same mixture) since 2014. Details of the tillage, fertilisation and biodynamic preparations factors are given in Table 1. All biodynamic preparations listed in Table 1 were sourced from a farmers’ group, all members of which are located within Switzerland and who jointly produce preparations according to Steiner^19^. In order to compare the effect of preparations to the untreated reference, the same feedstock of slurry was filled in two tanks and fresh manure piled to two piles for composting. At the day of manure delivery, 2 g of solid compost preparations BD502 to BD506 were each mixed with some manure and thrown into holes bored into the compost pile. 2 ml of liquid BD507 was stirred with 2.5 L of rain water and manually spread over the pile. The same was done for the slurry tank. Both composts were turned once and preparations added a second time to the biodynamic pile. In addition, field preparation BD500 was applied twice a year in spring and autumn and BD501 at least 3 times a year during crop growth with a backpack sprayer to biodynamic plots. Thereby, 25 g of BD500 was stirred in 10 L of heated (37 °C) rain water in the afternoon and similarly 1 g of BD501 in 10 L in the early morning on the day of application. Choice of application date based on planetary configuration. As the farm converted to biodynamic in 2010, manure was imported from an organic dairy farm close by (<10 km). Since manure source and composition differed between years, fertilisation intensity varied slightly between rotations with a mean total nitrogen input intensity of 1.0 livestock units ha^−1^.

**Table 1:** Management summary for the Frick long-term trial specified for the three management factors (2002–2018).

	Reference system	Alternative system
**Tillage**	**Ploughing (CT)**	**Reduced tillage (RT)**
Annual crops	Annual full inversion 15–18 cm with a mouldboard plough	1./2. Rotation: annual shallow mixing to 5 cm (Rototiller, “Stoppelhobel”) and loosening to 15 cm after wheat (“WEcoDyn” chisel)
		3. Rotation: annual shallow non-inversion to 5–10 cm (“WEcoDyn” chisel)
Seedbed	Rototiller, 5 cm	Rototiller, 5 cm
Ley establishment	Stubble tillage 5 cm and seedbed preparation with Rototiller 5 cm	Stubble tillage 5 cm and seedbed preparation with Rototiller 5 cm
Ley termination	Full inversion 15–18 cm just before seeding (February 2008 before silage maize, October 2013 before winter wheat)	September 2007: “Stoppelhobel” 5–8 cm, “WEcoDyn” 15 cm, seeding of a winter pea intercrop that was mulched and incorporated (5 cm) before seeding of silage maize in May 2008; details in Krauss, *et al*.^53^
		September 2013: “Stoppelhobel” 5–8 cm, “WEcoDyn” 8–10 cm just before seeding of winter wheat in October
Equipment	Various mouldboard ploughs, “Rototiller” - horizontally rotating harrow (Rau company, D-73235 Weilheim, Germany)	“Stoppelhobel” - skim plough with a wheel for working depth adjustment (Zobel Stahlbau, D-74585 Rot am See, Germany); “WEcoDyn” - chisel plough with flat goose sweeps, (Friedrich Wenz GmbH, D-77963 Schwanau, Germany),
**Fertilisation**	**Slurry (SL)**	**Manure compost** + **slurry (MC)**
Type	Liquid cattle slurry	Liquid cattle slurry, 0.5 times the rate of SL, plus composted cattle manure
Annual average fertilisation intensity: 1 livestock unit (LU) ha^−1^ based on total N input Annual average input (kg ha^−1^ a^−1^):		
Ntot/Nmin	101.0/43.2	104.3/27.5
P/K/Mg	20.3/156.5/19.1	26.6/169.5/27.3
OM	1705.8	2074.1
**Biodynamic prep**.	**Without**	**With**
	No application	BD500 – cattle manure, BD501 – silica, BD502 – *Achillea millefolium* L., BD503 – *Matricaria recutita* L., BD504 *Urtica dioica* L., BD505 – *Quercus robur* L., BD506 – *Taraxacum officinale*, Wiggers, BD507 – *Valeriana officinalis* L.
**Weed control**	Spring-tine weeder in all annual crops; Interrow cultivator in silage maize and sunflower (1–2 passes)	

Ntot – total nitrogen, Nmin – mineral nitrogen, P – phosphorus, K – potassium, Mg – magnesium, OM – organic matter.

### Soil analyses

The topsoil was sampled regularly every 3–4 years (2002, 2005, 2008, 2011, 2015, 2018) and the following parameters analysed: pH, soil organic carbon (SOC), nutrient contents, microbial biomass and microbial activity. Eight (minimum) to twelve soil samples per plot were taken manually, randomly across the plot, with a soil auger to gain enough soil for analysis and archive samples. These subsamples were each divided into 0–10 cm and 10–20 cm soil increments. Increments were then pooled per plot. pH was measured in a suspension of air-dried soil with H_2_O_dest_ (1:10, w/v). SOC was analysed by Walkley-Black wet oxidation of 1 g dry soil in 20 ml concentrated H_2_SO_4_ and 25 ml 2 M K_2_Cr_2_O_7_. Plant available soil nutrients (P, K, Mg) were extracted with ammonia acetate-EDTA. P was determined spectrophotometrically and K and Mg by atom absorption spectrometry. Microbial biomass C and N (Cmic, Nmic) were assessed by chloroform fumigation extraction of 20 g dry soil with 80 ml of a 0.5 M K_2_SO_4_ solution (1:4, w/v) as described in detail by Fließbach, *et al*.^20^ and subsequent analysis with a TOC/TnB analyser. Dehydrogenase (DHA) activity was determined according to Tabatabai^21^ incubating 5 g dry soil with triphenyl-tetrazolium chloride (TTC). The reaction product triphenylformazan (TPF) was extracted with acetone and determined with a spectrophotometer at 546 nm.

Data on earthworm abundance and the biomass of functional microbial groups assessed by the extraction of phospholipid fatty acids (PLFA) were taken from Kuntz, *et al*.^22^ where the methods are described in detail. The same refers to the arbuscular mycorrhizal fungi spore density assessment according to Säle, *et al*.^23^ and functional microbial genes determined by DNA extraction and subsequent qPCR analysis as described in Krauss, *et al*.^24^.

### Yields, crop nutrients and weed assessment

Crop and weed assessments were all performed in the core 8 × 8 m of each plot. For yield determination of cereal grain yield and total grass-clover biomass, a strip of 7 × 1.5 m or 7 × 1.9 m was harvested with a specialised plot combine harvester or a flail mower respectively. Straw yields of cereals and total biomass yields of silage maize and sunflower were assessed manually by cutting plants in two 0.5 × 0.5 m (cereals) and two 2 x row space (0.75 m in maize, 0.5 m in sunflower) subplots per plot. Plant and grain material was immediately dried at 60 °C. After grinding, nitrogen was analysed by Kjehldahl digestion and the nutrients P, K and Mg by HCl extraction of 600 °C incinerated ash and subsequent analysis with a spectrophotometer (P) and atom absorption spectrometry (K, Mg). Since 2014, N has been determined by dry combustion and P, K and Mg by ICP-MS determination after HCl extraction. Dry matter analysis at 105 °C finally allowed for dry matter yield calculation.

Weed cover in annual crops was estimated visually in two subplots of 0.5 × 0.5 m (cereals) or of 2 m × row space (0.75 m in maize, 0.5 m in sunflower). Within the subplot, total weed leaf area covering the ground surface was estimated by eye in a range of 0 to 100% as described in Sans, *et al*.^25^. Weed biomass was determined by removing all plants within the subplot and conducting subsequent dry matter analysis. Soil samples were taken in the slurry fertilised plots (SL) without biodynamic preparations for weed seed bank analysis in November 2015. Soil of four pseudoreplicates per plot and layer (0–7, 7–14, 14–21 cm) was sieved to 5 mm and placed in trays in a non-heated glasshouse over a period of eight months during the spelt season in 2015/2016. Trays were irrigated from the bottom if necessary, regularly mixed and covered with a net to avoid predation. Every four weeks in winter and every second week during spring and summer, emerged seedlings were counted, determined at species level and removed according to the protocol of Bàrberi and Lo Cascio^26^. Seedling emergence was very low at the end, indicating that most seedlings were registered.

### Nutrient balances

To estimate the nutrient balances of N, P and K over three rotations, fertiliser inputs were calculated against the exported nutrients in harvested products. For the N balance, estimated inputs from biologically fixed N were additionally included.

Biological nitrogen fixation (BNF, kg ha^−1^) by clover during the grass-clover ley periods was estimated according to Høgh-Jensen, *et al*.^27^:

1 $${\rm{BNF}}={{\rm{DM}}}_{{\rm{legume}}}\ast {\rm{N}} \% \,\ast \,{P}_{fix}\ast (1+{{\rm{P}}}_{\text{root}+\text{stubble}}+{{\rm{P}}}_{{\rm{transsoil}}}+{{\rm{P}}}_{{\rm{immobile}}})$$

where dry matter biomass of the legume (DM_legume_, kg ha^−1^) was calculated as the product of measured clover cover (%) and the total grass-clover biomass for each cut. Fixed values include: N% = mean N concentrations of red and white clover (3.4%), P_fix_ = fixed N_2_ as proportion of total N in the shoot dry matter (88%), P_root + stubble_ = fixed N_2_ in the root and stubble as proportion of totally fixed shoot N (0.25), P_transsoil_ = below-ground transfer of fixed legume N_2_ in the grass in mixtures (0.05) and P_immobile_ = fixed N_2_ immobilised in the organic soil pool (0.6 for clayey soils). N% and P_fix_ were taken from means between red and white clover samples measured in BIODYN (2^nd^ fertilisation level) in the study of Oberson, *et al*.^28^ which derive from a long-term field trial in close proximity to the Frick trial under similar management conditions. The values for P_root + stubble_, P_transsoil_ and P_immobile_ derive from Jensen, *et al*.^29^ for cut 1–2 years old grass-red clover and grass-white clover swards in clayey soils. BNF estimation resulted in 56.8 kg N fixed from the atmosphere per ton legume dry matter yield. N fixed by green manure legume mixtures after winter wheat was not assessed and could not be considered. Only the winter pea green manure in 2008 was sampled for biomass and N content and thus included in the N balance.

### Data analysis

All data analyses were run in R^30^ using the “nlme” package^31^ for statistics. Evolution of soil indicators, average yields and weed cover during 15 years were analysed with a repeated measures ANOVA (lme function) with Year and trial factors as fixed and Block as random effect. Temporal autocorrelation was addressed with a compound symmetry correlation on plot level nested in Year. Cmic, DHA and weed cover were log and P, K and yields square root transformed. To meet homoscedasticity, variance covariates (varIdent) encompassed Block or Block*Tillage. For all ANOVAs with single year data, trial factors nested in Blocks were included as random factors. Weed seed distribution was assessed with a negative binominal model (glm.nb function).

## Results

### Soil quality

**Figure 1:**
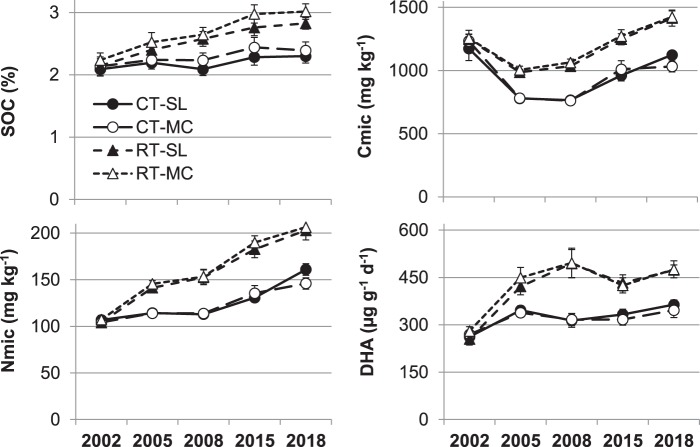
Development of soil organic carbon (SOC), soil microbial biomass C and N (Cmic, Nmic) and dehydrogenase activity (DHA) in the 0–10 cm soil layer from 2002 to 2018. Symbols represent means and error bars standard errors. Tillage factors are ploughing (CT) and reduced tillage (RT). Fertilisation includes a slurry system (SL) and a system with manure compost and slurry (MC).

**Figure 2:**
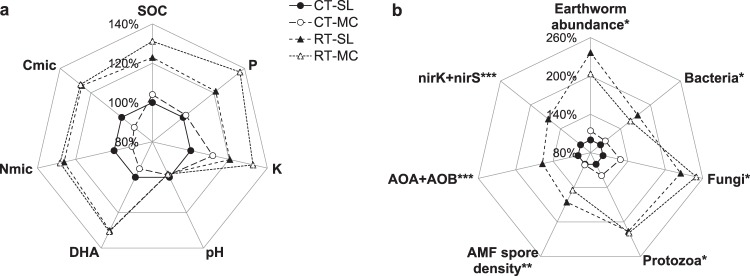
Mean relative differences (%) between alternative treatments to the standard treatment CT-SL (=100%) in the 0–10 cm soil layer of (**a**) soil biochemical indicators sampled in 2018 including pH, soil organic carbon (SOC), microbial biomass C and N (Cmic, Nmic), dehydrogenase activity (DHA) and plant available nutrients (P, K) (data pooled across biodynamic preparations, n = 8) and (**b**) soil biological indicators published in *Kuntz, *et al*.^22^ including total earthworm abundance and microbial groups determined by phospholipid fatty acid (PLFA) analysis (n = 4), **Säle, *et al*.^23^ including arbuscular mycorrhizal fungi (AMF) spore density (n = 4) and ***Krauss, *et al*.^24^ including rRNA data (nitrification and denitrification functional genes) (n = 3). Absolute results and detailed ANOVA analysis for 2a) are shown in Table S1 in the Supplement. Please note the different scales in (**a**,**b**).

Reducing tillage depth and intensity by converting from ploughing to shallow non-inversion tillage significantly increased indicators of soil quality in the topsoil layer of 0–10 cm: The increasing drift between tillage systems during 15 years is shown in Fig. 1; absolute values are influenced by the cumulative tillage effect but also by crop and weather conditions in the year of sampling. The drift is confirmed by the significant interaction of years and tillage system (Table 2). In 2018, relative differences between tillage systems cumulated in 25% higher soil organic carbon, 32% higher microbial biomass and 34% higher dehydrogenase activity with reduced tillage compared to ploughing (Fig. 2a, Table S1 Supplement). In parallel, PLFA analysis by Kuntz, *et al*.^22^ in 2011 found that earthworm and microbial abundance of fungi and protozoa more than doubled with reduced tillage while bacteria increased by only about 60% (Fig. 2b). Arbuscular mycorrhizal spore density measured by Säle, *et al*.^23^ in 2009 and nitrifiers (AOA/AOB) and denitrifiers (nirK/nirS, but not nosZ (not shown)) determined by Krauss, *et al*.^24^ in 2013 also increased with reduced tillage within a range of 40–60%. Phosphorus and potassium contents enriched in the topsoil layer compared to ploughing (P + 28%, K + 20%, Fig. 2a) while the magnesium content was unaffected (Table S1 Supplement). In contrast, pH decreased slightly with reduced tillage over time with a significant difference of 0.9 pH units in 2018 (Fig. 2a, Table S1 Supplement).

**Table 2:** Evolution of topsoil chemical and microbial soil quality indicators across the period of 2002 to 2018 tested with a repeated measures ANOVA (F-values and levels of significance, no indication – not significant, ^†^p < 0.1, *p < 0.05, **p < 0.01, ***p < 0.001) for long-term impacts of tillage, fertilisation and biodynamic preparations in 0–10 and 10–20 cm soil layers.

	pH	SOC	Cmic	Nmic	DHA	P	K
**0–10 cm**							
Year (Y)	228.1***	227.6***	278.1***	262.57***	106.7***	64.7***	715.6***
Tillage (T)	8.31**	169.6***	111.9***	214.75***	202.5**	102.7***	212.4***
Fertilisation (F)	1.28	30.1***	2.95†	0.61	0.01	0.17	43.9***
Biodyn. Prep. (P)	1.70	0.01	3.19†	4.46*	0.24	11.8**	1.01
Y x T	11.4***	53.5***	54.0***	34.46***	28.0***	53.3***	41.0***
Y x F	0.54	2.76*	1.07	1.03	0.70	2.52*	13.7***
Y x P	1.91	1.74	0.57	1.69	0.93	7.44***	0.75
**10–20 cm**							
Year (Y)	104.3***	22.8***	96.7***	67.22***	147.4***	45.9***	489.7***
Tillage (T)	3.26	2.64	13.8***	12.45**	27.7***	0.64	11.6**
Fertilisation (F)	2.21	3.21	1.74	0.21	0.01	1.74	20.2***
Biodyn. Prep.(P)	5.45*	0.01	0.10	0.05	0.01	0.10	3.39†
Y x T	0.95	2.66*	10.2***	8.68***	14.6***	0.40	3.31*
Y x F	0.50	0.24	0.47	0.46	3.36*	1.08	5.63***
Y x P	9.99***	1.32	2.59*	0.99	0.47	1.48	0.86

Data of all sampled years (2002, 2005, 2008, 2015, 2018) were considered. Indicators include: pH, soil organic carbon (SOC), plant available phosphorus (P) and potassium (K), microbial biomass C and N (Cmic, Nmic) and dehydrogenase activity (DHA).

In the lower topsoil layer (10–20 cm), the change in soil quality indicators between tillage systems was only persistent regarding increased microbial biomass (+15%) and dehydrogenase activity (+9%) in reduced tilled plots. In contrast, K content decreased by 6%. All other indicators were similar between tillage systems in this layer (Table S1 Supplement).

The additional application of manure compost while reducing the amount of slurry also increased the soil organic carbon (+6%), P (+8%, not significant) and K (+11%) content in 0–10 cm compared to pure slurry fertilisation (Table S1 Supplement). This was more pronounced in reduced-tillage soil than ploughed soil (Fig. 2a). In contrast, the Mg content decreased by 12–15%. Microbial indicators were not significantly affected by fertilisation (Fig. 2a,b).

Compared to the reference system in this trial (CT-SL), a stepwise enrichment of soil organic carbon, fungal abundance and P and K content in the 0–10 cm topsoil layer can be observed from CT-SL to CT-MC to RT-SL to RT-MC (Fig. 2). Calculating the stratification ratio between the 0–10 cm and the 10–20 cm layer indicates redistribution of soil biochemical indicators within the topsoil profile (Fig. S2 Supplement). Reduced tillage clearly increased stratification in nearly all assessed indicators compared to ploughing, with ratios ranging from 1.1 to 3.7 displaying enrichment in the surface layer. The stratification ratio was highest with regard to fungi and protozoa. Nitrifiers (AOA, AOB) were more abundant in the lower soil layer, as indicated by a stratification ratio below one.

Regarding biodynamic preparations, there were significant differences regarding P content in 0–10 cm and pH in 10–20 cm, as well as a trend in terms of microbial biomass over time (Table 2). In 2018 and in both soil layers, 4–6% lower microbial biomass and 6–7% lower P contents were detected in plots treated with biodynamic preparations while pH was similar in both treated and untreated plots (Table S1 Supplement). pH was lower in plots treated with biodynamic preparations in 2015 only (data not shown), which may explain the significant year effect in Table 2.

### Agronomic performance

**Figure 3:**
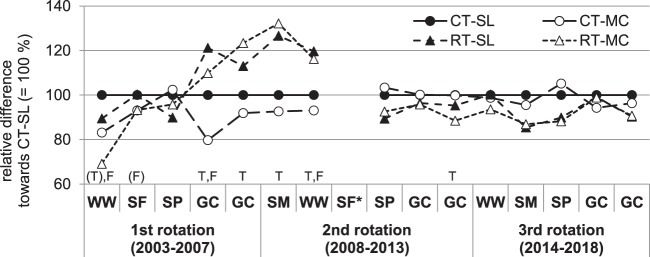
Mean relative difference (%) of marketable yields from 2003 to 2018 between alternative tillage and fertilisation treatments, with CT-SL set to 100%. Lines are drawn to facilitate readability. Biodynamic preparations were pooled. Crops include WW - winter wheat, SF – sunflower, SP – spelt, GC – grass-clover and SM – silage maize. SF* – yield loss in 2010. Significant differences between tillage (T) and fertilisation (F) treatments as well as interactions are indicated in capital letters (p < 0.05, (p) <0.1). Absolute yields and detailed ANOVA analysis are shown in Table S2 in the Supplement.

Marketable yields varied considerably between crops and years (detailed data in Table S2, Supplement). Comparing alternative tillage and fertilisation systems with the reference CT-SL (=100%), reduced tillage mainly induced higher yields between 2006 and 2009 and slightly but insignificantly lower yields afterwards (Fig. 3). Yields as affected by fertilisation differed only in some years, with manure compost/slurry application (MC) resulting in lower yields than pure slurry application (SL). In 2010, there was a complete loss of sunflower yields in ploughed plots due to slugs. On average, relative marketable yields across all crops and years were 2% higher under reduced tillage compared to ploughing, with interannual variation clearly displayed by the significant Year/Tillage interaction (Table 3). Specifically, there was an average yield gap in winter (cereal) crops by −4% while spring crops in reduced tillage yielded an average 8% increase towards ploughing. Yields in MC were on average 3% lower than SL with significant Year/Fertilisation interactions in total and winter crop yields indicating that nutrient supply was less sufficient with MC than SL especially in cereals. Biodynamic preparations had no effect on marketable yields.

**Table 3:** Mean (standard deviation) of relative differences (%) in marketable yields and weed cover between tillage, fertilisation and biodynamic preparation treatments across 2003–2018.

*n (crop years)*		Marketable yield			Weed cover		
		Total	Winter crops	Spring crops	Total	Winter crops	Spring crops
		Relative difference in %					
		15	6	3	9	5	4
Tillage	RT vs. CT (CT = 100%)	102 (16) %	96 (14) %	108 (24) %	273 (205) %	200 (79) %	365 (289) %
Fertilisation	MC vs. SL (MC = 100%)	97 (6) %	97 (9) %	97 (3) %	104 (14) %	102 (17) %	106 (12) %
Biodyn. Prep.	with vs. without (without = 100%)	99 (4) %	99 (5) %	97 (1) %	86 (13) %	87 (15) %	86 (12) %
**ANOVA**							
*denDF*		415	165	81	249	137	109
Year (Y)		1261.2***	746.7***	2130.4***	30.9***	3.76**	43.7***
Tillage (T)		4.17*	5.65*	12.9**	121.0***	91.3***	144.4***
Fertilisation (F)		1.98	1.88	0.02	1.20	0.69	1.74
Biodyn. Prep. (P)		0.03	0.00	0.76	0.25	0.57	0.11
YxT		22.6***	17.4***	72.5***	14.4***	5.80***	15.3***
YxF		3.47***	9.91***	0.85	0.44	0.38	0.12
YxP		0.57	1.04	0.12	0.37	0.46	0.08

Winter sown crops include winter wheat and spelt and spring sown crops include silage maize and sunflower. Grass-clover was only included in total yield analysis. Absolute yields per year are shown in Table S2 in the Supplement. Treatment differences were tested with an ANOVA (F-values and levels of significance, ns – not significant, †p < 0.1, *p < 0.05, **p > 0.01, ***p > 0.001).

Treatment effects on yields were reflected by an increased nutrient export with reduced tillage and pure slurry application (Table 4). Regarding the N balance, total N input was higher in the reduced tillage system due to an additional legume intercrop before maize in 2008 which was not introduced in the ploughing system. Alongside a higher total N input, P and K inputs were on average larger in MC than SL. In terms of surpluses, P was balanced overall while K and total N input was larger than their export. However, the N balance is not complete as inputs and exports of a mixed leguminous/non-leguminous intercrop after winter wheat in 2003, 2009 and 2014 could not be included in the calculation since data were missing. Biodynamic preparations had a minor effect on nutrient balances which was mainly introduced by the variation in fertiliser input that comes with the use of normal farm machinery.

**Table 4:** Mean total nitrogen (N), phosphorus (P) and potassium (K) balance across three crop rotations in kg ha^−1^ year^−1^.

			Total N balance				P balance			K balance		
			Fertiliser Input	BNF Input	Export	**Surplus**	Fertiliser Input	Export	**Surplus**	Fertiliser Input	Export	**Surplus**
Tillage		RT	102.7	94.4	142.6	**54.4**	23.5	25.7	**−2.2**	163.0	139.8	**23.2**
		CT	102.7	88.3	135.2	**55.8**	23.5	24.6	**−1.1**	163.0	138.5	**24.5**
Fertilisation		MC	104.3	91.9	137.2	**59.0**	26.6	24.6	**2.0**	169.5	137.7	**31.8**
		SL	101.0	90.8	140.6	**51.2**	20.3	25.7	**−5.4**	156.5	140.6	**15.9**
Biodynamic Prep.		without	103.8	91.2	138.9	**56.2**	23.1	25.3	**−2.2**	162.2	138.9	**23.3**
		with	101.5	91.5	138.9	**54.0**	23.8	25.0	**−1.1**	163.8	139.3	**24.4**

Weed cover was assessed visually in all arable crops. There was a high variation between crops and years with a mean increase in weed infestation of 173% under reduced tillage compared to ploughing (Table 3). Weed infestation under reduced tillage was higher in spring crops like maize and sunflower (+265%) than winter (cereal) crops (+100%). There was a slight but insignificant decrease of 14% when biodynamic preparations were applied. In arable crops, weed biomass was often determined in addition (data not shown). Weed cover and weed biomass correlated positively (log(weed cover) = −0.11 + 0.69*log(weed biomass), DF 126, F-value 49.5, p < 0.0001, adjusted R^2^ 0.28).

**Figure 4:**
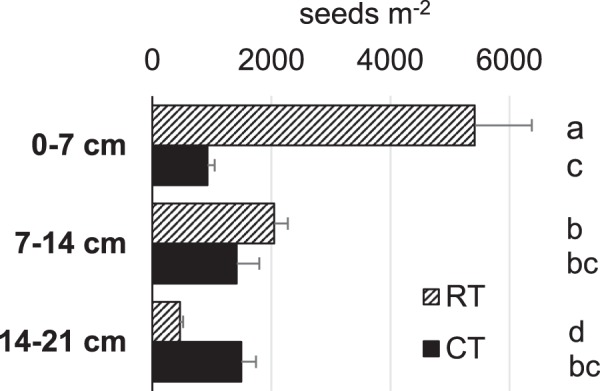
Weed seed bank in the 0–7, 7–14 and 14–21 cm soil layer of tillage systems in slurry fertilised (SL) plots without biodynamic preparations. Bars (means, standard errors) represent number of seeds m^−2^ in ploughed (CT, black) and reduced tillage (RT, dashed) plots, letters indicate significant differences (p < 0.05).

Analysis of the weed seed bank in 2016 gives an idea of the potential weed pressure that accumulated over the years (Fig. 4). While weeds were homogeneously distributed over the 0–20 cm soil layer under ploughing, reduced tillage clearly induced a significant stratification with the largest amounts of weed seeds in the uppermost 7 cm. In total, about a two-fold increase in seeds was observed in reduced tillage plots (7936 seeds m^−2^) compared to ploughing (3846 seeds m^−2^) in the 0–20 cm topsoil layer, which was highly significant (ANOVA, F-value 24.5, p < 0.0001).

## Discussion

By far the greatest impact on soil quality was induced by conversion from ploughing to reduced tillage across nearly all the measured chemical and biological soil indicators. Solid manure application and the application of biodynamic preparations resulted in less pronounced effects on a selection of indicators. Altering the soil habitat mechanically thus had a greater impact than additional external inputs.

Reduced tillage led to an enrichment of soil organic carbon (SOC) and nutrients, and also of labile carbon^32,33^ in the still tilled top 10 cm layer, while there was no difference to ploughing in the 10–20 cm layer. Pronounced stratification with reduced tillage was found for SOC stocks until 50 cm soil depth^34^. Such an increased stratification within the soil profile when comparing conservation with conventional tillage has been widely described for SOC stocks^35,36^ and nutrient stocks^37,38^. Luo, *et al*.^35^ explained this in terms of a lack of soil mixing that promotes topsoil accumulation, and a lack of mechanical soil loosening in lower layers that causes reduced root growth and thus a lower C input by root matter and exudates. Lack of mixing is most likely also the driver for topsoil nutrient accumulation. A trend towards decreased SOC and nutrient stocks in deeper soil layers as found by Luo, *et al*.^35^ and Neugschwandtner, *et al*.^37^ could not be found in this trial. With an average of 45% clay containing mainly illites, but also smectites and vermiculites, it is most likely that clay minerals contributed to SOC stabilisation in our case. Reactive clay minerals are known to stabilise microbial-derived^39^ and root-derived^40^ SOC by physico-chemical protection. In this trial, clay minerals still appear to be undersaturated^32^. Alongside SOC, a higher aggregate stability^41^ and hydrostructural stability^32^ in the reduced tillage system has already been measured in the same trial. It is most likely that other soil physical properties are also improved, at least in the SOC-rich and microbially active topsoil.

Less tillage disturbance seems to impact the living conditions of soil organisms, as found by, e.g., Helgason, *et al*.^42^ who detected less bacterial stress markers in no-till soils, a higher microbial abundance in general and an altered microbial community composition. The latter has been widely found^43–45^ and can be confirmed fully by measurements made in this trial, even with reduced instead of no tillage. We have found a constantly higher microbial biomass in both the 0–10 and 10–20 cm soil layers and a higher dehydrogenase activity in the topsoil over 15 years. Microbial biomass correlated well with SOC content^34^, which indicates improved food availability. A community shift has also be seen: reduced tillage favoured gram negative bacteria, fungi and protozoa in general^22^ and arbuscular mycorrhizal fungi^23,46^ and nitrifiers and denitrifiers^24^ specifically. A lower bacterial to fungi ratio suggests a more fungal-based community under reduced tillage^22^, a finding supported by existing knowledge^39,43,47^. The higher fungal abundance may be linked to less disturbance of the hyphal network^48^ but also to the decrease in pH in reduced tillage plots, as Rousk, *et al*.^49^ found higher growth rates of fungi with lower pH. In our trial, a decline in pH in reduced tillage plots was already detected after six years^50^ and has been confirmed by the most recent measurements. This may be an outcome of a more undisturbed pedogenesis, since the topsoil is not mixed with lower layers in contrast to ploughing. Finally, earthworms and especially juveniles and cocoons and endogenic species have been found to profit from reduced tillage in this trial^18,22^ and from conservation tillage in general^51^. Reducing tillage intensity has therefore largely improved the topsoil quality at this site, while altering the soil habitat and enhancing ecological functions like climate adaptation, biodiversity preservation and soil erosion control. At this site reduced tillage has potential as a climate mitigation measure, as greenhouse gas emissions were similar to ploughing in two cropping seasons and SOC stocks (0–50 cm) were enriched compared to ploughing^34^. The higher microbial biomass and activity, however, has been shown to pose the risk of higher short-term greenhouse gas emissions when microbes are stimulated, e.g. after tillage operations^34^. Careful management is hence needed. It is questionable whether reduced tillage in organic farming reduces fuel use, as has been reported for no tillage with herbicide use^52^. In our practical experience in Switzerland, we have found that more passes are often required to achieve weed control or to successfully remove a grass-clover ley^53^. This may outbalance the benefit of SOC sequestration and requires future research and LCA assessment for a full picture.

When comparing a pure liquid cattle slurry amendment (SL) to mixed application of composted cattle manure and reduced amounts of slurry (MC), increased contents of SOC and K and also a tendency towards increased P in MC can be explained by the higher input with solid manure application. Regarding SOC, an increase of 6% was achieved with a 22% increase in organic matter input over 15 years. Unlike tillage, the enrichment in SOC only resulted in a slight increase in microbial biomass, which was significant only in some years^22^ while dehydrogenase activity was not enhanced^18,50^. PLFA analysis did not reveal differences in community composition^22^. Yet nitrous oxide peak emissions found to be higher in MC than SL after tillage operations indicate an impact of long-term composted manure application on microbial activity, at least of certain functional groups^34^. This was unexpected, as molecular analyses in the DOK trial had revealed a distinct effect by the application of manure compost (biodynamic system) on community composition^54^.

The addition of biodynamic preparations led to a tendency towards decreasing P content in the 0–10 cm layer, which was not prevalent at trial start. This was already indicated in Gadermaier, *et al*.^50^ in 2008 and significant in 2018. The tendency cannot be explained by higher crop exports. P concentrations were also not increased at 10–20 cm, which could indicate migration to greater depths. This observation thus remains unexplained. In contrast, a slightly lower pH and microbial biomass were already recorded at trial start, which may indicate spatial heterogeneity. In 2018, microbial biomass C and N were significantly lower in plots treated with biodynamic preparations, which was not reported in similar studies so far^55–58^. A higher Cmic/Nmic ratio in 2005 indicated a shift in microbial communities^50^ which seemed to be a single effect as it was not confirmed in later soil analyses. This is in line with soil microbial studies that similarly did not find effects of biodynamic preparations on soil microbial communities^55,59^. Regarding microbial activity, Zaller and Kopke^58^ reported lower basal respiration and metabolic coefficients with biodynamic preparations, whereas in this and other studies, enzyme activities (e.g. dehydrogenase activity in our case) or respiration measurements yielded no treatment differences^55,57,59^. There is hence not a clear effect of biodynamic preparations on soil quality. It is more likely that manure compost has more impact on soil quality in biodynamic farming than biodynamic preparations, as suggested by Hartmann, *et al*.^54^.

Yields were mainly affected by tillage system in the Frick trial. In the first rotation, yields in the reduced tillage system were overall lower than under ploughing. Such decreases during conversion can be explained by soil structure issues and a reduced N supply^60^, although such a trend could not be found in a meta-analysis of a larger data collection^61^. Clover did not establish in ploughed plots due to dry weather conditions during ley establishment in 2006 in the end of the first rotation. In consequence, precrop effects differed between tillage systems for the following arable crops. In combination with the additional N from pea green manure, yields of maize (2008) and wheat (2009) were 34% and 22% greater in the reduced tillage system^53^. This was mirrored in the overall N balance by higher N inputs via biological N_2_ fixation of legumes and the consequently higher N export in reduced tillage. Minor differences between tillage systems in terms of P and K balance indicate that N was the more important driver. In the last two rotation periods, yields were continuously lower in the reduced tillage system, which corresponds better to the more general finding that organic reduced tillage reduces yields by 7.6% on average^61^. Cooper, *et al*.^61^ found that N supply and weeds are major constraints; our findings underscore this. N supply differences between tillage treatments were not only affected by N_2_ fixation but also by spring mineralisation in our trial. When spring was dry and warm, as seen in 2014, wheat yields in weed-cleaned subplots were higher in reduced tillage due to more active soil mineralisation. Yet, the high weed infestation impaired this yield potential^62^. In other, wetter and cold springs, cereal yields were lower with reduced tillage (e.g. 2016). Compaction of the old plough layer below the new tillage depth (in our case 10–20 cm, Krauss, *et al*.^34^ Supplement) most likely decreased yields despite the better soil structure in the topsoil layer^32,41^ by impairing root growth and N mineralisation. Soil aeration without soil inversion may help to improve water and air flow to deeper soil depths^63^. Weeds were found to be problematic in this trial, especially perennial weeds^25,64^ and mostly in spring row crops that are less competitive against weeds. The enrichment of weed seeds in the topsoil further points to future problems. Overall, yield stability seems to be less guaranteed by continuous reduced tillage compared to ploughing as a consequence of altered weed incidence, water, air and N supply. Strategic improvements of existing rotations with green manures, occasional soil aeration and strategic ploughing in situations of high weed infestation may be suitable measures to achieve conservation outcomes with a long-term perspective.

In terms of fertiliser type, yields tended to be higher in slurry fertilised fields, especially in cereals. Available N most likely drove yield differences, as the total N and organic matter input was higher in the manure compost/slurry system. However, this fertilisation effect was only prevalent in three out of 15 crop years. This indicates that reducing the amount of slurry and complementing with composted manure maintains sufficient nutrient supply and yields while not worsening weed pressure and, moreover, enhances soil quality.

Biodynamic preparations did not affect nutrient balances and yields as seen elsewhere for arable crops^16,58^, although higher yields for potatoes, for instance, were found to be associated with the application of preparations^16^. An overall trend is thus difficult to discern, and is not actually expected as biodynamic preparations are thought to balance yields^65^. A slightly lower weed pressure with the application of biodynamic preparations in the reduced tillage system may reflect such a balancing influence; to our knowledge, such an outcome has not been reported so far.

## Supplementary information

Supplementary Information.

## Data Availability

The datasets generated and/or analysed during the current study are available from the corresponding author on request.
