# Supplementary material for: Enhanced soil quality with reduced tillage and solid manures in organic farming – a synthesis of 15 years
Source: Sci Rep. 2020 Mar 10;10:4403. doi: 10.1038/s41598-020-61320-8 (PMC7064577; doi:10.1038/s41598-020-61320-8)
Supplement: Supplementary file 1 — Supplementary Information. [file 41598_2020_61320_MOESM1_ESM.pdf]

Enhanced soil quality with reduced tillage and solid manures in organic farming – a synthesis of 15 years

Maike Krauss, Alfred Berner, Frédéric Perrochet, Robert Frei, Urs Niggli, Paul Mäder

Department of Soil Sciences, Research Institute of Organic Agriculture (FiBL), 5070 Frick, Switzerland

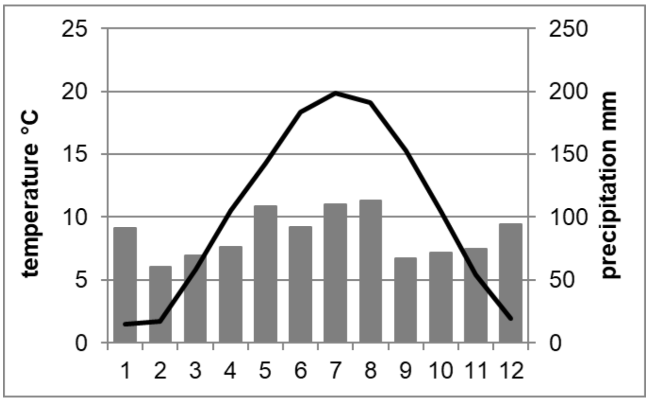

Figure S1. Average monthly precipitation (mm, bars) and temperature (°C, line) in 2003-2018 in Frick.

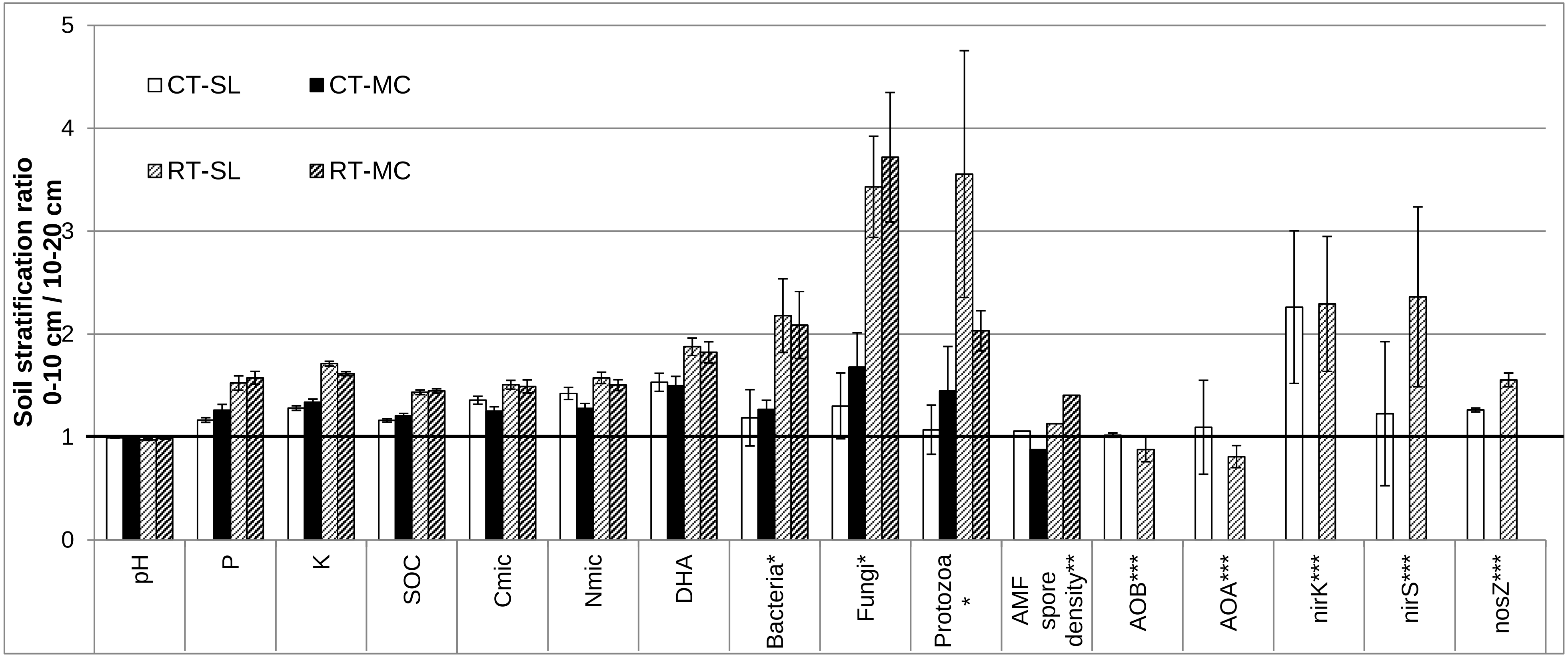

Figure S2. Soil stratification ratio between the 0-10 and 10-20 cm soil layer of soil chemical and biological indicators. Tillage factors are ploughing (CT) and reduced tillage (RT). Fertilisation includes a slurry system (SL) and a system with manure compost and slurry (MC). Soil biochemical data were sampled in 2018. Those data were pooled for biodynamic preparations (n = 8). *Abundance of bacteria, fungi and protozoa derive from PLFA analysis (n=4) from Kuntz et al. (2013), **arbuscular mycorrhizal (AMF) spore densities (n=4) from Säle et al. (2015) and ***nitrifiers and denitrifier rRNA data (n=3) from Krauss et al. (2017) include only data from plots without biodynamic preparations.

Table S1. Chemical and biological soil parameters sampled in 2018 in the 0-10 cm and 10-20 cm soil layer. Means (standard deviation) are displayed. Treatment differences were tested with a linear mixed effect model (F-values and levels of significance, (*)p<0.1, *p<0.05, **p>0.01, ***p>0.001). P – phosphorous, K – potassium, Mg – magnesium, SOC – soil organic carbon, Cmic/Nmic – microbial biomass C and N, DHA – dehydrogenase activity. CT – ploughing, RT – reduced tillage, SL – slurry, MC – manure compost/slurry.

			0-10 cm								10-20 cm							
Till.	Fert.	Prep.	pH	P	K	Mg	SOC	Cmic	Nmic	DHA	pH	P	K	Mg	SOC	Cmic	Nmic	DHA
			(H_2O)		(mg kg^{-1} DM)		(%)	(mg kg^{-1} DM)		(μg g^{-1} DM d^{-1})	(H_2O)		(mg kg^{-1} DM)		(%)	(mg kg^{-1} DM)		(μg g^{-1} DM d^{-1})
CT	MC	without	7.27	118.6	605.9	1495.7	2.37	1097.9	155.7	367.6	7.36	100.0	454.3	1543.8	1.98	840.8	119.2	238.9
			(0.27)	(25.6)	(23.4)	(727.2)	(0.44)	(80.9)	(15.2)	(36.8)	(0.22)	(35.2)	(31.3)	(792.0)	(0.30)	(105.5)	(16.8)	(58.9)
CT	MC	with	7.25	108.7	601.6	1740.0	2.42	966.2	136.2	325.0	7.37	86.3	449.0	1784.8	1.97	813.8	110.9	234.5
			(0.29)	(28.8)	(45.6)	(1045.0)	(0.39)	(127.7)	(13.8)	(87.2)	(0.16)	(28.1)	(33.7)	(1031.1)	(0.26)	(88.3)	(17.8)	(73.0)
CT	SL	without	7.38	116.4	559.9	1912.4	2.26	1148.0	165.6	354.0	7.45	99.6	427.2	1936.2	1.98	820.3	112.7	251.0
			(0.20)	(6.0)	(48.1)	(908.5)	(0.29)	(4.7)	(3.9)	(28.2)	(0.17)	(10.8)	(41.0)	(882.9)	(0.25)	(49.3)	(12.8)	(35.2)
CT	SL	with	7.32	106.9	522.8	1697.6	2.33	1099.8	156.3	373.8	7.35	92.8	418.2	1698.8	1.97	839.0	114.1	236.5
			(0.27)	(21.3)	(33.6)	(762.8)	(0.37)	(102.9)	(25.7)	(56.7)	(0.21)	(19.3)	(23.6)	(829.0)	(0.28)	(45.7)	(7.8)	(66.2)
RT	MC	without	7.26	158.3	722.6	1359.2	3.04	1401.3	204.9	498.5	7.29	103.4	441.0	1407.8	2.14	1006.8	143.3	284.9
			(0.20)	(17.1)	(38.6)	(603.1)	(0.38)	(76.2)	(10.7)	(58.2)	(0.16)	(22.3)	(32.5)	(728.6)	(0.33)	(109.4)	(15.3)	(33.9)
RT	MC	with	7.18	147.7	712.4	1446.4	2.99	1447.7	207.7	452.6	7.36	93.6	447.8	1571.0	2.04	918.5	132.1	248.7
			(0.26)	(19.0)	(52.9)	(749.9)	(0.38)	(186.5)	(35.1)	(94.8)	(0.29)	(15.6)	(34.2)	(928.7)	(0.32)	(109.6)	(19.4)	(69.9)
RT	SL	without	7.18	136.5	655.0	1802.1	2.90	1501.7	214.5	458.2	7.40	89.4	374.7	1845.5	1.98	970.2	134.9	259.9
			(0.29)	(10.0)	(22.4)	(846.5)	(0.29)	(202.8)	(31.4)	(37.0)	(0.16)	(12.7)	(20.5)	(874.7)	(0.20)	(87.6)	(17.0)	(38.1)
RT	SL	with	7.24	134.4	649.5	1699.1	2.75	1328.9	191.3	488.5	7.40	91.6	387.1	1694.7	1.97	906.8	123.6	251.4
			(0.25)	(18.1)	(50.1)	(870.1)	(0.26)	(121.5)	(24.8)	(52.5)	(0.31)	(23.6)	(23.9)	(872.0)	(0.29)	(80.0)	(18.5)	(44.9)
ANOVA																		
Tillage (T)			12.77*	24.89*	104.71**	1.73 ^{ns}	97.22**	61.57**	74.64**	58.74**	0.24 ^{ns}	0.00 ^{ns}	5.37 ^{ns}	0.84 ^{ns}	1.21 ^{ns}	25.57*	20.06*	1.35 ^{ns}
Fertilisation (F)			2.80 ^{ns}	2.38 ^{ns}	33.79**	13.47*	7.23*	1.53 ^{ns}	1.40 ^{ns}	0.24 ^{ns}	1.86 ^{ns}	0.08 ^{ns}	19.05**	11.56*	1.17 ^{ns}	0.20 ^{ns}	2.05 ^{ns}	0.05 ^{ns}
Biodyn. Prep. (P)			1.16 ^{ns}	8.51*	1.91 ^{ns}	0.00 ^{ns}	0.95 ^{ns}	5.25*	6.32*	0.60 ^{ns}	0.01 ^{ns}	3.22(*)	0.03 ^{ns}	0.00 ^{ns}	2.76 ^{ns}	6.86*	8.35*	2.82 ^{ns}
T x F			3.90(*)	1.52 ^{ns}	0.02 ^{ns}	1.22 ^{ns}	0.85 ^{ns}	2.28 ^{ns}	3.54 ^{ns}	0.40 ^{ns}	0.27 ^{ns}	0.42 ^{ns}	2.67 ^{ns}	1.00 ^{ns}	1.33 ^{ns}	0.30 ^{ns}	0.95 ^{ns}	0.92 ^{ns}
T x P			0.46 ^{ns}	0.39 ^{ns}	0.39 ^{ns}	0.04 ^{ns}	12.23*	0.16 ^{ns}	0.18 ^{ns}	0.02 ^{ns}	0.68 ^{ns}	0.68 ^{ns}	1.15 ^{ns}	0.00 ^{ns}	1.57 ^{ns}	5.51*	2.33 ^{ns}	0.47 ^{ns}
F x P			1.05 ^{ns}	0.66 ^{ns}	0.46 ^{ns}	7.70*	0.71 ^{ns}	1.03 ^{ns}	0.65 ^{ns}	7.88*	1.19 ^{ns}	1.43 ^{ns}	0.00 ^{ns}	11.72**	1.69 ^{ns}	1.34 ^{ns}	0.86 ^{ns}	0.22 ^{ns}

Table S2. Marketable yields of three crop rotation periods in the Frick trial between 2003 and 2018. Mean yields (standard deviation) are displayed in t dry matter ha^{-1} and include grain yields for winter wheat (WW), sunflower (SF) and spelt (SP) and total biomass yields for grass-clover (GC) and silage maize (SM). *In 2010, only sunflower biomass yields can be given due to slug invasion. Treatment differences were tested with an ANOVA (F-values and levels of significance, (*)p<0.1, *p<0.05, **p>0.01, ***p>0.001). CT – ploughing, RT – reduced tillage, SL – slurry, MC – manure compost/slurry.

			1st rotation					2nd rotation						3rd rotation				
year			2003	2004	2005	2006	2007	2008	2009	2010	2011	2012	2013	2014	2015	2016	2017	2018
crop			WW	SF	SP	GC	GC	SM	WW	SF*	SP	GC	GC	WW	SM	SP	GC	GC
Till.	Fert.	Prep.																
CT	MC	without	4.46	3.04	2.34	6.85	7.28	11.31	3.33	9.35	2.31	8.72	11.27	4.32	10.57	1.23	10.86	9.21
			(0.68)	(0.27)	(0.53)	(0.89)	(0.76)	(1.14)	(0.31)	(0.95)	(0.18)	(0.69)	(1.4)	(0.37)	(2.56)	(0.19)	(1.04)	(1.73)
CT	MC	with	4.13	3.01	2.46	6.48	7.65	12.29	3.26	8.55	2.30	8.44	12.28	4.45	10.24	1.45	9.41	8.73
			(1.51)	(0.51)	(0.55)	(0.52)	(0.92)	(1.03)	(0.25)	(1.06)	(0.17)	(0.87)	(0.96)	(0.28)	(1.06)	(0.13)	(1.13)	(1.06)
CT	SL	without	5.50	3.35	2.55	8.26	8.65	12.64	3.55	8.04	2.17	8.59	11.67	4.49	11.16	1.28	10.46	9.19
			(0.23)	(0.21)	(0.11)	(0.69)	(0.63)	(1.19)	(0.2)	(0.15)	(0.19)	(0.59)	(0.71)	(0.24)	(1.59)	(0.28)	(1.56)	(2.06)
CT	SL	with	4.84	3.15	2.15	8.45	7.60	12.83	3.53	8.82	2.29	8.55	11.92	4.40	10.63	1.27	11.04	9.44
			(1.18)	(0.2)	(0.31)	(0.53)	(1.56)	(0.4)	(0.31)	(0.58)	(0.45)	(0.65)	(1.19)	(0.38)	(1.42)	(0.26)	(1.21)	(2.75)
RT	MC	without	3.58	3.17	2.22	9.34	9.99	17.10	4.03	6.30	1.98	8.40	10.34	4.23	9.21	1.18	10.66	8.38
			(1.19)	(0.39)	(0.52)	(0.8)	(0.86)	(0.87)	(0.24)	(0.89)	(0.28)	(0.79)	(0.5)	(0.45)	(0.67)	(0.58)	(1.21)	(0.93)
RT	MC	with	3.57	2.89	2.28	9.03	10.05	16.57	4.20	7.36	2.15	8.01	10.54	4.08	9.72	1.07	10.59	8.53
			(1.18)	(0.64)	(0.72)	(0.46)	(0.62)	(1.08)	(0.25)	(1.18)	(0.31)	(0.67)	(0.92)	(0.48)	(0.6)	(0.52)	(1.78)	(1.90)
RT	SL	without	4.78	3.30	2.24	10.10	9.07	16.95	4.20	6.77	2.05	8.12	11.17	4.62	9.62	1.04	10.43	8.20
			(0.2)	(0.2)	(0.4)	(0.81)	(1)	(1.04)	(0.08)	(0.64)	(0.21)	(0.38)	(0.48)	(0.14)	(0.64)	(0.17)	(0.90)	1.00
RT	SL	with	4.47	3.22	1.98	10.15	9.29	15.31	4.27	7.44	1.94	8.39	11.32	4.29	8.99	1.25	10.89	8.63
			(0.43)	(0.26)	(0.33)	(0.27)	(2.1)	(1.52)	(0.14)	(0.86)	(0.14)	(0.74)	(0.61)	(0.28)	(0.94)	(0.42)	(1.21)	(1.29)
ANOVA																		
Tillage (T)			6.93†	0.01ns	1.25ns	70.3**	32.1*	94.4**	120.2**	17.5*	5.40ns	0.98ns	10.3*	0.58ns	4.85ns	0.81ns	0.73ns	1.67ns
Fertilisation (F)			15.9**	4.31†	0.32ns	63.5***	0.08ns	0.07ns	7.56*	0.23ns	0.92ns	0.005ns	1.95ns	1.46ns	0.08ns	0.05ns	1.92ns	0.08ns
Biodyn. Preparations (P)			3.87†	4.27ns	2.35ns	0.45ns	0.11ns	0.68ns	0.37ns	2.92ns	0.42ns	0.62ns	2.61ns	2.38ns	0.52ns	1.15ns	0.28ns	0.04ns
T x F			0.14ns	0.0004ns	0.06ns	5.14†	5.49†	3.58ns	0.85ns	2.51ns	0.0004ns	0.02ns	1.77ns	0.69ns	0.32ns	0.20ns	1.54ns	0.12ns
T x P			1.02ns	0.21ns	0.08ns	0.02ns	0.56ns	7.53*	1.44ns	3.02ns	0.04ns	0.12ns	0.84ns	3.14ns	0.3ns	0.11ns	1.80ns	0.21ns
F x P			0.93ns	0.01ns	7.21*	1.96ns	0.96ns	2.42ns	0.04ns	1.40ns	0.40ns	2.47ns	0.65ns	2.00ns	0.94ns	0.12ns	7.34*	0.33ns
